# Secondary compound hypothesis revisited: Selected plant secondary metabolites promote bacterial degradation of *cis*-1,2-dichloroethylene (cDCE)

**DOI:** 10.1038/s41598-017-07760-1

**Published:** 2017-08-16

**Authors:** Serena Fraraccio, Michal Strejcek, Iva Dolinova, Tomas Macek, Ondrej Uhlik

**Affiliations:** 1 University of Chemistry and Technology, Prague, Faculty of Food and Biochemical Technology, Department of Biochemistry and Microbiology, Prague, Czech Republic; 2 Technical University of Liberec, Liberec, Czech Republic

## Abstract

*Cis*-1,2-dichloroethylene (cDCE), which is a common hazardous compound, often accumulates during incomplete reductive dechlorination of higher chlorinated ethenes (CEs) at contaminated sites. Simple monoaromatics, such as toluene and phenol, have been proven to induce biotransformation of cDCE in microbial communities incapable of cDCE degradation in the absence of other carbon sources. The goal of this microcosm-based laboratory study was to discover non-toxic natural monoaromatic secondary plant metabolites (SPMEs) that could enhance cDCE degradation in a similar manner to toluene and phenol. Eight SPMEs were selected on the basis of their monoaromatic molecular structure and widespread occurrence in nature. The suitability of the SPMEs chosen to support bacterial growth and to promote cDCE degradation was evaluated in aerobic microbial cultures enriched from cDCE-contaminated soil in the presence of each SPME tested and cDCE. Significant cDCE depletions were achieved in cultures enriched on acetophenone, phenethyl alcohol, *p*-hydroxybenzoic acid and *trans*-cinnamic acid. 16S rRNA gene sequence analysis of each microbial community revealed ubiquitous enrichment of bacteria affiliated with the genera *Cupriavidus*, *Rhodococcus*, *Burkholderia*, *Acinetobacter* and *Pseudomonas*. Our results provide further confirmation of the previously stated secondary compound hypothesis that plant metabolites released into the rhizosphere can trigger biodegradation of environmental pollutants, including cDCE.

## Introduction

Chlorinated ethenes (CEs) are common environmental pollutants due to their intense involvement in anthropogenic activities and improper waste disposal^1^. Increasing concerns regarding CE toxicity and their adverse effects on human health (https://www.epa.gov/toxics-release-inventory-tri-program) have led to much stricter environmental regulations^2^ which have limited the use of CEs and tightened the rules on their cleanup in the environment. In this context, microbial bioremediation represents a more promising and cost-effective strategy as compared to traditional physical-chemical methods^3^. In the subsurface, CEs with higher chlorination levels easily undergo anaerobic biodegradation to less chlorinated CEs but rarely to non-chlorinated compounds^3, 4^. Despite the possible involvement of many different types of bacteria in this process^5, 6^, only members of the genus *Dehalococcoides* have been described as capable of complete anaerobic dechlorination to ethene^7–9^. Consequently, incomplete anaerobic degradation of CEs occurs in many sites, resulting in the accumulation of biodegradation intermediates, such as *cis*-1,2-dichloroethylene (cDCE) and vinyl chloride (VC), which is more toxic and carcinogenic^10, 11﻿^ than higher CEs.

Microbial degradation of lower CEs can be achieved under aerobic conditions. The degradation is initiated by di- or monooxygenase enzymes^12^. Several studies have demonstrated that many microbial species are able to use VC as a sole carbon source and to achieve its complete mineralization^5, 12^. However, aerobic growth has been far less commonly observed on cDCE, which has only been thoroughly described for *Polaromonas* sp. strain JS666^13–15^. Little additional evidence of direct aerobic cDCE degradation has been reported for isolates affiliated with *Acinetobacter* sp., *Achromobacter* sp., *Bacillus* sp., *Pseudomonas* sp.^16^ or with mixed microbial cultures retrieved from a stream bed sediment^17^ and from CE-contaminated groundwater^18^. On the other hand, aerobic microbial degradation of cDCE can occur via aerobic cometabolism (AC), a process driven by oxygenase enzymes which have relaxed substrate specificity^19, 20^. In this case, cDCE is fortuitously oxidized in the presence of a growth-supporting substrate that stimulates enzymatic activity of the oxygenase^21^. Growth-supporting substrates, which have been described in relation to cDCE AC processes, are ethene^22^, methane^23, 24^, propane^25^, butane^26^, propene^27^, ammonia^28^ and some less chlorinated hydrocarbons such as VC^29, 30^. In addition, monoaromatic compounds, such as phenol, toluene and benzene, can efficiently enhance aerobic cometabolic mineralization of cDCE^31, 32^. Some difficulties with respect to AC implementation arise from the introduction of some of the above-mentioned substrates into the environment mostly due to their toxicity^33^.

Secondary plant metabolites (SPMEs), which enter the soil via different routes, including root exudation and litter decomposition, constitute an important source of nutrients for soil bacteria and play naturally established roles in plant-microbe communication and chemotactic regulation of soil microbial colonization^34, 35^. Extensive research has demonstrated that plant-derived compounds also constitute a precious source of promising alternative substrates in order to trigger biodegradative pathways for xenobiotics, including polychlorinated biphenyls (PCBs) and polyaromatic hydrocarbons (PAHs)^36–39^. This phenomenon is referred to as the “secondary compound hypothesis”^40^. In the present study, we sought monoaromatic SPMEs which could be used to assist the degradation of cDCE, possibly through aerobic cometabolism, in a manner similar to that described in relation to phenol and toluene. To our knowledge, this proof-of-concept study is the first to explore the possibility of cDCE degradation induced by monoaromatic SPMEs, which were used to support microbial growth through a laboratory-enrichment cultivation procedure.

## Results

### Degradation of cDCE by toluene- and phenol-exposed microbial community

**Figure 1:**
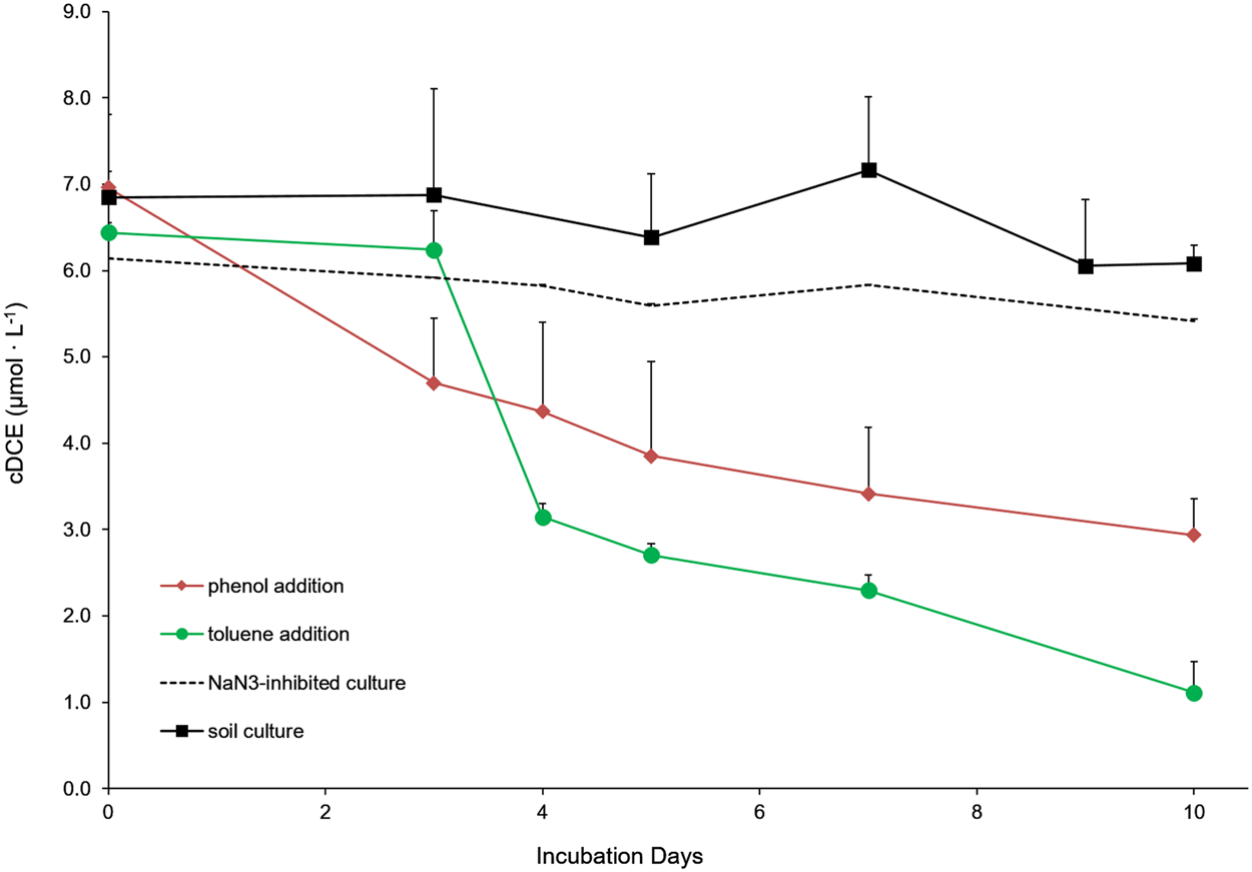
Average depletions of cDCE in the water phase of the soil microbial cultures in the presence of only cDCE (filled squares) after the addition of toluene (closed circles) or phenol (closed rectangles). The dashed line indicates the control sodium azide-inhibited phenol-grown cultures. The error bars indicate the standard deviation among three biological replicates prepared per each condition.

At the initial stage of this study, the microbial community in the contaminated soil was shown not to be capable of cDCE degradation when cDCE was provided as the sole carbon source during the first 10 days of cultivation (Fig. 1). Measurement of cDCE concentrations in the culture headspaces performed up to the third month of cultivation revealed the persistence of cDCE in the liquid cultures (with approximately an 11% of variability among all measurements).

On the other hand, when exposed to toluene and phenol, microbial suspensions extracted from soil successfully degraded cDCE over a period of 10 days (Fig. 1). Higher cDCE depletion levels were achieved in the presence of toluene, with a 82.78% ± 3.73 percentage points (pp) removal of the initial amount despite an initial lag phase of approximately three days (Fig. 1); in the presence of phenol, depletion was 57.89% ± 3.43 pp. Much lower cDCE loss (about 11.8% pp) was observed over the same period of time in phenol-supplied control cultures inhibited with sodium azide (Fig. 1), suggesting that cDCE depletion in toluene- and phenol-supplied cultures was attributable to microbial activity.

### Degradation of cDCE by SPME-enriched microbial cultures

**Figure 2:**
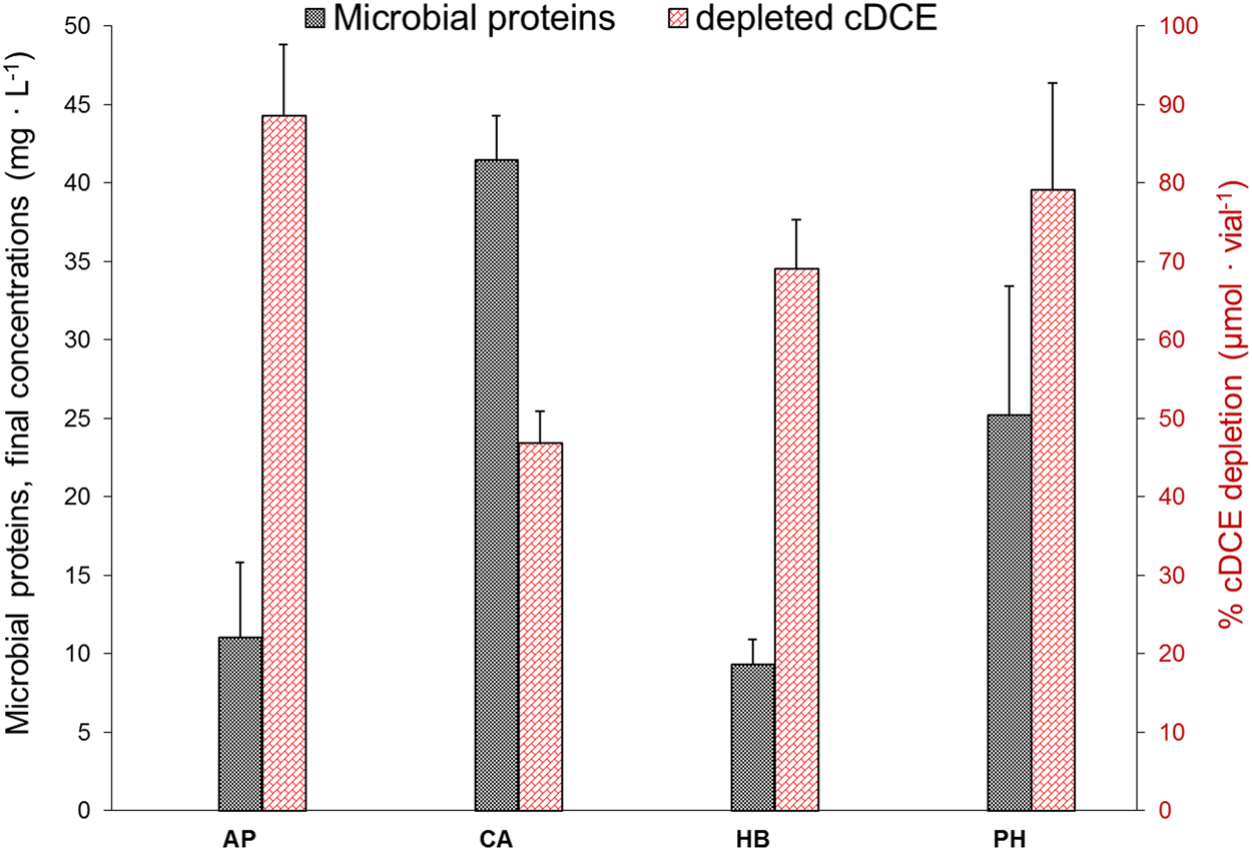
Microbial concentration, estimated as microbial protein content, and depletion of cDCE (% out of the initial amount) after 10 incubation days for the cultures enriched on 50 mg∙L^−1^ acetophenone (**AP**), *trans*-cinnamic acid (**CA**), *p*-hydroxybenzoic acid (**HB**) and phenethyl alcohol (**PH**). The error bars indicate the standard deviation calculated on the basis of three biological replicates.

**Figure 3:**
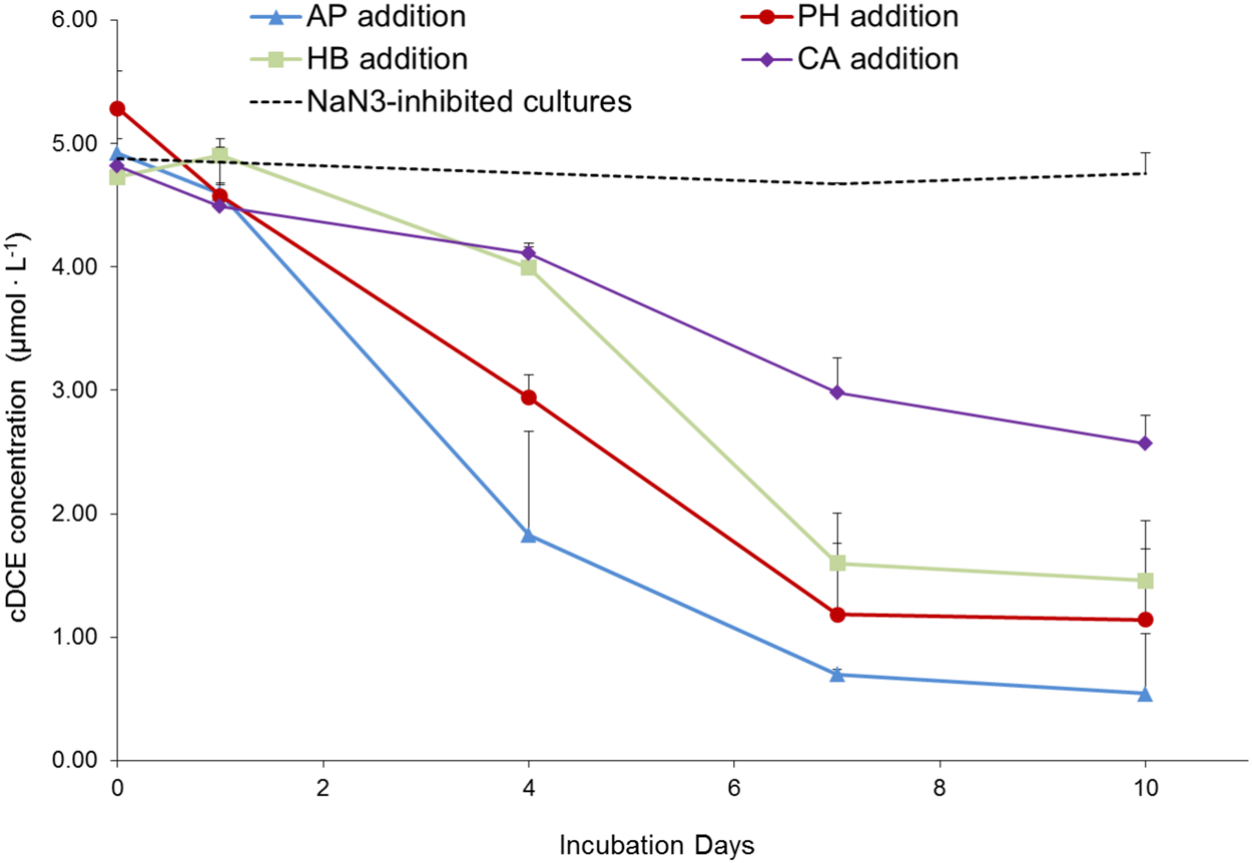
Average depletion of cDCE over time in the aqueous phase for the cultures enriched on acetophenone (**AP**); *trans*-cinnamic acid (**CA**); *p*-hydroxybenzoic acid (**HB**); and phenethyl alcohol (**PH**) (three biological replicates were used per each condition). Dashed line indicates the over-time cDCE concentration in sodium azide-inhibited cultures grown on acetophenone and *p*-hydroxybenzoic acid (two biological replicates were used per control condition). The error bars indicate the standard deviation among biological replicates.

The biodegradation of cDCE was first evaluated for eight microbial cultures where the primary carbon sources were represented by different monoaromatic SPMEs. After approximately a month of cultivation, cDCE depletion over a period of 10 days was monitored for all cultures. The highest cDCE depletion levels were attained in the presence of acetophenone (10.14%), phenethyl alcohol (8%), *p*-hydroxybenzoic acid (9.34%) and *trans*-cinnamic acid (7.11%). These four cultures were subjected to further enrichment through serial sub-culturing passages, during which substrate depletion, microbial growth and cDCE degradation were evaluated. At the final enrichment stage, all of the SPMEs tested were totally consumed within the first 18 to 25 hours of incubation, despite a lag phase of approximately 10 hours. The measurements of microbial concentrations at the end of the final sub-culturing stage indicated that microbial growth was more effectively supported by *trans*-cinnamic acid and phenethyl alcohol (Fig. 2). At the same time, significant cDCE depletion was achieved in the presence of acetophenone (88.59% ± 9.08 pp of the initial amount of cDCE), phenethyl alcohol (79.18% ± 13.53 pp), *p*-hydroxybenzoic acid (69.05% ± 6.30 pp) and *trans*-cinnamic acid (46.82% ± 4.10 pp) (Fig. 2). When the concentration of cDCE in the liquid phase was plotted over time (Fig. 3), significant cDCE degradation was found to occur after the first day of cultivation, once the SPME substrates were already consumed, and to slow down considerably after seven days. Negligible cDCE loss (below 5% pp) was observed in sodium azide-inhibited cultures prepared with *p*-hydroxybenzoic acid as carbon source. Transformation yields of cDCE per SPME, expressed as µmol_(cDCE)_∙mmol_(SPME)_^−1^, were: 16.38 ± 1.55 for acetophenone, 16.32 ± 1.91 for *p*-hydroxybenzoic acid, 10.53 ± 1.31 for phenethyl alcohol and 9.80 ± 0.76 for *trans*-cinnamic acid.

### Structural diversity of SPME-enriched microbial populations

**Figure 4:**
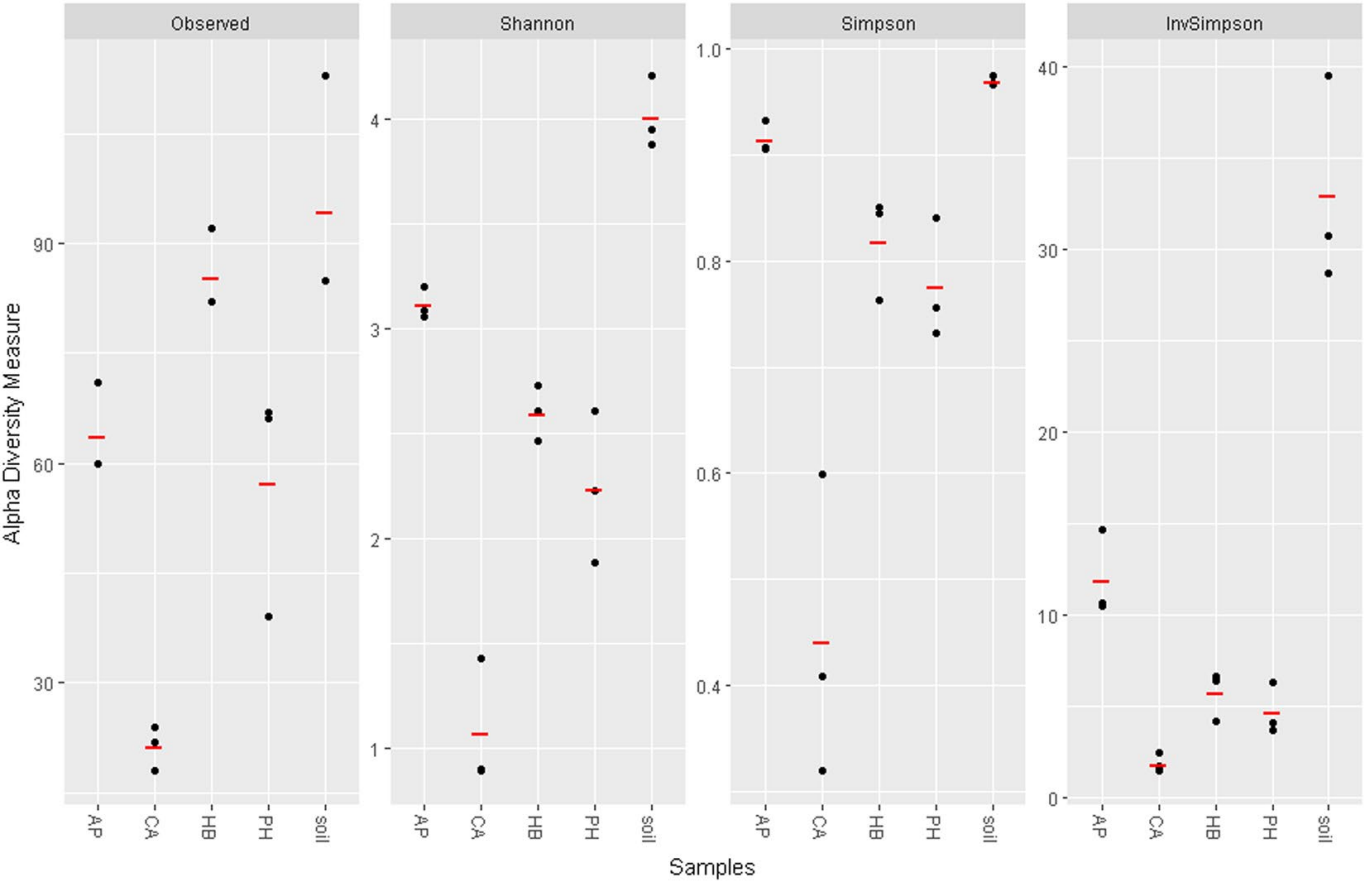
Number of observed sequence variants and diversity indexes of the microbial communities from the original soil or microbial populations enriched on each SPME (acetophenone **AP**; *trans*-cinnamic acid **CA**; *p*-hydroxybenzoic acid **HB**; phenethyl alcohol **PH**; each dot represents one biological replica, mean values are shown as red bars). Abbreviations: Observed – number of observed sequence variants, Shannon – Shannon-Wiener index, Simpson – Simpson’s index of diversity (1–*D*), InvSimpson – Inverse Simpson’s index (1/*D*).

**Figure 5:**
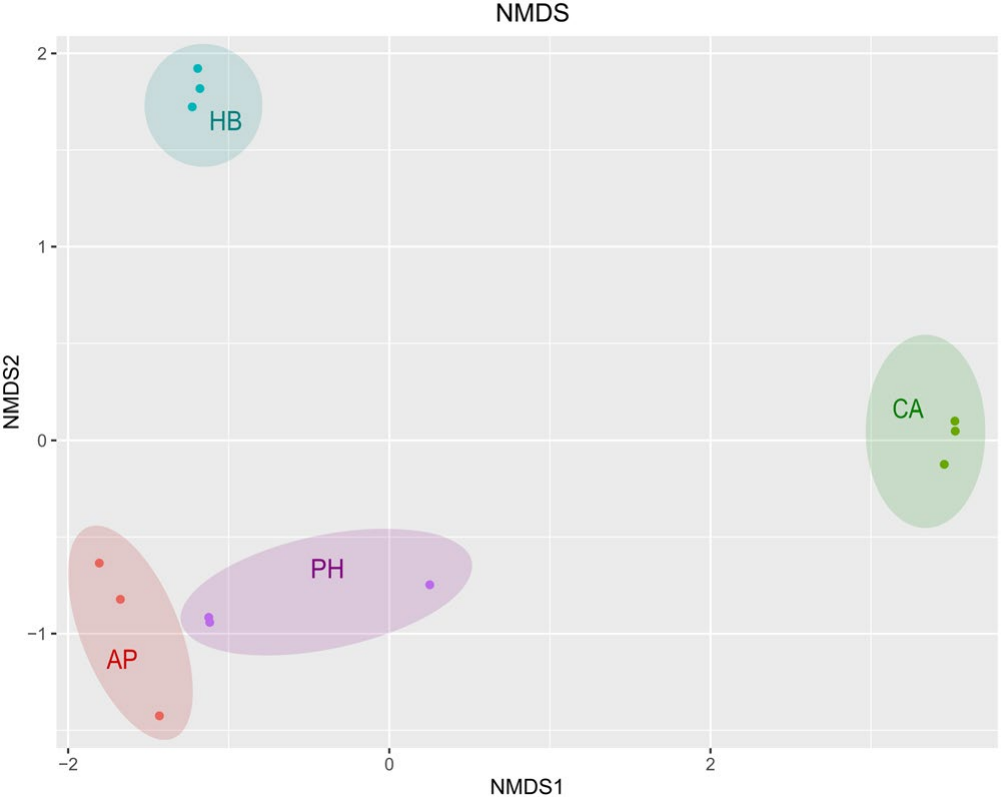
Non-metric multidimensional scaling (NMDS) plot for the enriched microbial populations (acetophenone **AP**; *trans*-cinnamic acid **CA**; *p*-hydroxybenzoic acid **HB**; phenethyl alcohol **PH**). Each biological replicate is represented by a dot.

The highest levels of richness, as assessed by the number of sequence variants observed, or phylogenetic diversity, as assessed by the Shannon, Simpson and reciprocal Simpson diversity indexes, were clearly observed for the initial soil microbial community (Fig. 4). Remarkable differences were observed between the SPME-enriched microbial populations, where the highest diversity indexes were found in acetophenone-enriched cultures, followed by *p*-hydroxybenzoic acid and phenethyl alcohol-amended cultures, while *trans*-cinnamic-enriched populations were characterized by the lowest diversity indexes (Fig. 4). In order to highlight the structural differences between the SPME-enriched microbial populations, a non-metric multidimensional scaling (NMDS) analysis was carried out. This showed that the cultures enriched on *p*-hydroxybenzoic acid and *trans*-cinnamic acid formed distinct clusters, while cultures enriched on acetophenone and phenethyl alcohol were more related (Fig. 5).

### Phylogenetic analysis of the most abundant taxa

The relative abundance profiles of the thirty most abundant sequence variants in the treatments were evaluated. A ubiquitous enrichment of taxa within *Proteobacteria* and *Actinobacteria* occurred in the enrichment cultures (Table 1). The most commonly detected sequence variants amongst *Betaproteobacteria* clustered with *Burkholderiaceae*, with *Cupriavidus* being the most abundant taxon in cultures enriched on *trans*-cinnamic acid and phenethyl alcohol and quite abundant in cultures enriched on the other SMPEs. Members of the families *Comamonadaceae* (*Simplicispira*, unclassified *Comamonadaceae*) and *Oxalobacteraceae* (*Massilia*) were also profusely detected. *Gammaproteobacteria* were mainly represented by *Xanthomonadaceae* (genera *Dokdonella*, *Pseudoxanthomonas, Rhodanobacter*) and the genera *Pseudomonas, Acinetobacter* and *Legionella*. *Alphaproteobacteria* were more abundant in acetophenone-enriched cultures, which particularly included *Devosia* and *Sphingopyxis*. Acetophenone-enriched populations were characterized by a number of additional phylogenetic classes, such as *Verrucomicrobiae, Alphaproteobacteria*, *Flavobacteria*, *Sphingobacteria*, *Actinobacteria* (*Rhodococcus*) and *Planctomycetia*, as well as an enrichment of unclassified bacteria. A significant enrichment of *Deltaproteobacteria* (mainly *Bdellovibrio*) was observed in the presence of *p*-hydroxybenzoic acid and also in the other enriched microbial populations (Table 1

**Table 1:** Phylogenetic affiliation of the thirty most abundant enriched bacterial sequence variants.

Phylogenetic affiliations^*a*^	Closest RDP SeqMatch Type Strain	Score^*b*^	Accession No.	Treatment [relative abundance]			
				AP	CA	HB	PH
*Gammaproteobacteria*							
* Legionellaceae*							
*** Legionella***	*Legionella pneumophila* Losangelos-1	0.963	HQ287902	0.077	ND	ND	ND
* Moraxellaceae*							
*** Acinetobacter***	*Acinetobacter calcoaceticus* NCCB22016, *Acinetobacter pittii* LMG 1035	1.000	AJ888983, HQ180184	ND	0.024	ND	ND
*** Acinetobacter***	*Acinetobacter johnsonii* ATCC 17909	1.000	Z93440	0.022	ND	ND	ND
* Pseudomonadaceae*							
*** Pseudomonas***	*P. fulva* AJ 2129, *P. flavescens* B62, *P. argentinensis* CH01, *P. benzenivorans* DSM 8628	1.000	AB046996, U01916, AY691188, FM208263	0.001	ND	0.013	0.006
*** Pseudomonas***	*P. corrugata* ATCC29736, *P. kilonensis* 520-20, *P. vancouverens* DhA-51, *P. jessenii* CIP 105274, *P. moorei* RW10, *P. mohnii* IpA-2	1.000	D84012, AJ292426, AJ011507, AF068259, AM293566, AM293567	ND	0.024	ND	ND
* Xanthomonadaceae*							
*** Dokdonella***	*Dokdonella immobilis* LM 2-5	0.968	FJ455531	**0.167**	**0.007**	**0.001**	**0.117**
*** Rhodanobacter***	*Rhodanobacter caeni* MJ01	0.995	GQ250431	0.010	0.035	ND	< 0.001
*** Pseudoxanthomonas***	*Pseudoxanthomonas yeongjuensis* GR12-1	0.997	DQ438977	0.012	ND	0.002	0.006
unclassified	Unclassified bacterium	<0.800	—	0.033	ND	ND	0.012
*Betaproteobacteria*							
* Burkholderiaceae*							
*** Cupriavidus***	*Cupriavidus necator* ATCC 43291, *Cupriavidus basilensis* DSM 11853	1.000	AF191737, AF312022	**0.052**	**0.724**	**0.101**	**0.365**
*** Burkholderia***	*Burkholderia grimmiae* R27	0.989	JN256678	ND	ND	0.095	ND
*** Burkholderia***	*Burkholderia grimmiae* R27	0.995	JN256678	ND	ND	0.024	ND
* Comamonadaceae*							
*** Simplicispira***	*Simplicispira psychrophila* LMG 5408	1.000	AF078755	0.006	0.018	ND	0.146
*** Mitsuaria***	*Mitsuaria chitosanitabida* 3001	0.995	AB006851	0.016	ND	ND	0.005
* Oxalobacteraceae*							
*** Massilia***	*Massilia aerilata* 5516S-11, *Massilia tieshanensis* TS3	1.000	EF688526, HM130516	ND	ND	0.023	ND
*Alphaproteobacteria*							
* Hyphomicrobiaceae*							
*** Devosia***	*Devosia insulae* DS-56	1.000	EF012357	0.018	ND	0.005	0.006
*Sphingomonadaceae*							
*** Sphingopyxis***	*Sphingopyxis chilensis* S37, *Sphingopyxis panaciterrae* Gsoil 124	1.000	AF367204, AB245353	**0.012**	**0.001**	**0.008**	**0.001**
*Sphingobacteria*							
* Chitinophagaceae*							
*** Ferruginibacter***	*Ferruginibacter lapsinanis* HU1-HG42	0.952	FJ177532	ND	ND	ND	0.061
*** Ferruginibacter***	*Ferruginibacter lapsinanis* HU1-HG42	0.973	FJ177532	0.035	ND	ND	ND
*** Ferruginibacter***	*Ferruginibacter alkalilentus* HU1-GD23	0.955	FJ177530	0.024	ND	ND	ND
*** Sediminibacterium***	*Sediminibacterium salmoneum* NJ-44	0.965	EF407879	**0.017**	**<0.001**	**0.034**	**0.014**
*Sphingobacteriaceae*							
*** Solitalea***	*Solitalea koreensis* R2A36-4	0.912	EU787448	0.040	ND	ND	ND
*Deltaproteobacteria*							
* Bdellovibrionaceae*							
*** Bdellovibrio***	*Bdellovibrio bacteriovorus* HD 100	0.947	AJ292759	**0.001**	**0.002**	**0.372**	**0.001**
*** Bdellovibrio***	*Bdellovibrio bacteriovorus* HD 100	0.963	AJ292759	0.001	<0.001	0.106	ND
*Phaselicystidaceae*							
*** Phaselicystis***	*Phaselicystis flava* SBKo001	0.936	EU545827	0.016	ND	0.002	0.002
*Actinobacteria*							
* Nocardiaceae*							
*** Rhodococcus***	*Rhodococcus jostii* IFO16295	1.000	AB046357	**0.026**	**<0.001**	**<0.001**	**0.051**
*** Rhodococcus***	*Rhodococcus wratislaviensis* NCIMB 13082, *Rhodococcus koreensis* DNP505	1.000	Z37138, AF124342	0.029	ND	ND	0.042
*Flavobacteriia*							
* Flavobacteriaceae*							
*** Chryseobacterium***	*Chryseobacterium anthropi* NF 1366	0.984	AM982786	0.025	0.123	ND	0.003
*Bacteroidetes*							
* Bacteroidetes incertae sedis*							
*** Prolixibacter***	*Prolixibacter bellariivorans* F2	0.867	AY918928	0.061	ND	ND	ND
*Verrucomicrobiae*							
* Verrucomicrobiaceae*							
*** Prosthecobacter***	*Prosthecobacter fluviatilis* HAQ-1	0.981	AB305640	0.041	ND	ND	0.006

^*a*^Phylogenetic affiliations are based on Ribosomal Database Project (RDP) Classifier.

^*b*^Similarity reports the percent sequence identity over all pairwise comparable positions.

ND: Not detected.

). Interestingly, none of the enriched taxa were detected with frequencies of more than 0.001% in the original soil.

## Discussion

This proof-of-concept study demonstrates the possibility of cDCE aerobic degradation promoted by the presence of SPMEs used as carbon and energy sources during the enrichment of specific soil microbial populations. The initial microbial community extracted from CE-contaminated soil was shown to be incapable of cDCE biodegradation when exposed to cDCE without the addition of any other carbon and energy source.

Successful cDCE degradation was first achieved upon exposure of the soil microbial community to toluene or phenol (Fig. 1). A number of studies have demonstrated that cDCE biodegradation is attainable by using aerobic cometabolism with aromatic hydrocarbons such as toluene and phenol^41, 42^.

Promising bioremediation potential has often been ascribed to microorganisms associated with plant roots^43–46^. Some studies have even shown that, in polluted environments, the composition of root exudates can drive a selection of rhizosphere microbial populations with a greater capacity to degrade pollutants^47, 48^. Given the structural analogy between toluene, phenol and some monoaromatic SPMEs, we hypothesized that these SPMEs might promote the degradation of cDCE in a manner similar to that for toluene and phenol. Microbial growth on some plant metabolites has already been demonstrated with regard to the cDCE-assimilating bacterium *Polaromonas* sp. JS666 which is capable of growing on catechol, gentisate and salicylate^14^. Moreover, other plant compounds, including certain types of terpenes, have been used as growth-supporting substrates to enhance microbial degradation of other CEs^49^. The choice of the initially screened SPMEs depended on their monoaromatic structure and on the fact that they are widespread natural compounds produced by different plant species (details of the natural distribution of the SPMEs mentioned in this study are available from the U.S. Department of Agriculture at https://phytochem.nal.usda.gov). This study provides evidence that selected monoaromatic SPMEs can support microbial growth and cDCE depletion. In order to obtain enrichment cultures, the SPME concentrations used in this study were based on previous studies, in which same-class SPMEs were used during research on the cometabolic degradation of other xenobiotic compounds, including PCBs and trichloroethylene^49, 50^. At the final stage of enrichment, the presence and type of substrate were found to greatly influence cDCE degradation activity. According to the monitoring data (Fig. 3), cDCE depletion was limited during the first day of cultivation in the presence of acetophenone and zero in the presence of *p*-hydroxybenzoic acid. This might be due to substrate inhibition phenomena, which are frequently observed during cometabolism^51^. By contrast, cDCE depletion was detectable from the first day of incubation for cultures enriched on phenethyl alcohol and *trans*-cinnamic acid. In all enrichment cultures, cDCE degradation activity was notable until day 7 of cultivation and decreased considerably thereafter. The overall transformation of cDCE was more effectively driven by acetophenone-, *p*-hydroxybenzoic acid- and phenethyl alcohol-enriched microbial populations (Fig. 2) compared to that enriched on *trans*-cinnamic acid. In particular, the highest yields of cDCE removal per SPME used were observed for acetophenone- and *p*-hydroxybenzoic acid-enriched cultures. It is noteworthy that increased cDCE biodegradation activity took place in acetophenone- and *p*-hydroxybenzoic acid-enriched cultures, in which lower final microbial concentrations (Fig. 2) were observed along with higher alpha diversity indices for the enriched microbial populations (Fig. 4). In addition, a clear match was found between the increase in alpha diversity and biodegradative activity, expressed as cDCE transformation yields, which is in line with the finding that increased diversity is generally an indication of greater community resilience and is associated with a higher degree of bioremediation efficiency^52^. As highlighted by NMDS analysis, the enrichment of diverse microbial populations was strictly dependent on the type of SPME used. The two-dimensional NMDS plot in Fig. 5 shows how enrichment on *p*-hydroxybenzoic acid and *trans*-cinnamic acid shapes the microbial community in a very different way to that on acetophenone and phenethyl alcohol.

16S rRNA gene-based phylogenetic analysis revealed ubiquitous enrichment of several sequence variants affiliated with microbial taxa, which have been reported to act as degraders of many classes of environmental pollutants. These include members of the following genera: *Cupriavidus*, previously found to be involved in the degradation of phenolic and chloroaromatic compounds as well as toxic aromatic petroleum hydrocarbons^53^; *Rhodococcus*, whose members are notorious for their biodegradation capabilities, also involving chlorinated aliphatic hydrocarbons such as trichloroethylene and VC^54, 55^; *Sphingopyxis*, with *S. chilensis* being reported to be capable of growing on certain chlorophenolic compounds^56^; *Sediminibacterium*, whose member *S. salmoneum* was detected in a butane-enriched mixed microbial culture capable of aerobic cometabolic degradation of trichloroethylene^57^; *Dokdonella*, detected in the aqueous phase of a model wetland supplied with cDCE- and *trans-*DCE-contaminated water^58^; and *Pseudomonas*, whose members are known to contain di- and monooxygenases which can initiate cDCE oxidation upon induction of cometabolic growth on monoaromatic substrates^59, 60^. In this study, different pseudomonads were detected, including those closely related to *P. fulva*, *P. flavescens*, *P. benzenivorans* and *P. argentinensis* enriched in acetophenone, phenethyl alcohol and *p*-hydroxybenzoic acid cultures; and those associated with *P.corrugata, P. kilonensis* and *P. jessenii*, enriched in *trans*-cinnamic acid cultures. Furthermore, enrichment of several unclassified taxa of the family *Comamonadaceae* occurred in all enriched populations. All the most abundant taxa enriched on SPMEs were mostly below the detection limit in the initial microbial community, which provides further evidence that these taxa were involved in cDCE degradation, as the community initially present in the contaminated soil was not capable of cDCE degradation.

Growth on *p*-hydroxybenzoic acid was the only condition to raise the abundance levels of *Bdellovibrio* spp. (Table 1). A previous study has reported the enrichment of members of the family *Bdellovibrionaceae* in microbial communities, which occurred at a contaminated aquifer after toluene-based biostimulation experiments were performed on the *in situ* cometabolization of trichloroethylene^61^. However, *Bdellovibrio* spp. are known to parasitize other Gram-negative bacteria^62^, which may also explain their abundance in SPME-enriched populations. Other microbial sequence variants were exclusively observed in some of the SPME-enriched cultures; this was the case for *Acinetobacter johnsonii* detected in acetophenone cultures as well as *Acinetobacter calcoaceticus* and *Acinetobacter pittii* in *trans*-cinnamic acid cultures (Table 1). To date, a limited number of *Acinetobacter* spp. have been shown to participate in the degradation of pollutants such as diesel fuel hydrocarbons via alkane hydroxylase enzymes^63, 64^. 16S rRNA gene data analysis demonstrated that the type of SPME remarkably influenced the structure and composition of enriched microbial populations, which was a reflection of diverse cDCE degradative activities. However, the enrichment of microbial genera, such as *Cupriavidus*, *Burkholderia*, *Pseudomonas*, and *Rhodococcus* (Table 1), throughout the SPME-enriched cultures suggests that microorganisms affiliated with those genera might play a role in the degradation of cDCE.

In conclusion, this study provides evidence of enhanced cDCE degradation by microbial communities selected in the presence of SPMEs. Our results suggest that natural attenuation of cDCE in soil might occur as a result of the activity of plant-associated soil microorganisms. The study of SPME-stimulated microbial degradation of CEs could open up new avenues for the exploitation of SPMEs in biotechnology in areas such as the development of purpose-built phytoremediation techniques. In basic research terms, additional data are required on the fate of the original substrates – both SPMEs and cDCE – including the identity of microbial populations which assimilate them as well as the pathways used for their degradation. This could be done with the aid of parallel stable isotope probing experiments as shown elsewhere^65^. Furthermore, the isolation of cDCE-degrading pure microbial cultures could shed light on cDCE degradation mechanisms, including the molecular basis of SPME action, the genes/enzymes involved and their regulation.

## Materials and Methods

### Chemicals, culture medium and cultivation conditions

Liquid cDCE (97%) and all the SPMEs (purity higher than 97% for caffeic acid and vanillic acid, while higher than 99% for all the other SPMEs) used were obtained from Sigma-Aldrich (USA). Toluene was purchased from Penta (Czech Republic), and phenol (99.5%) was obtained from Fluka (USA). All other chemicals were of analytical grade. Liquid microbial cultures were prepared in an inorganic chloride-free minimal salt medium (MSM) with the following composition per liter of deionized water: Na_2_HPO_4_∙12H_2_O 11.0 g, KH_2_PO_4_ 2.7 g, (NH_4_)_2_SO_4_ 1 g, MgSO_4_∙7H_2_O 0.2 g, FeSO_4_∙7H_2_O 0.02 g and Ca(NO_3_)_2_ 0.03 g, pH 7. No vitamins or complex growth factors were added to the MSM. All the microbial cultures were aerobically established in 119 mL-serum vials (Sigma-Aldrich), sealed with teflon-lined butyl rubber septa (Sigma-Aldrich) and shaken on a rotary shaker at 130 rpm at 20 °C. The liquid phase to headspace (air) ratio in each vial was 1:3. Unless otherwise specified, all the experiments described were set up in biological triplicates.

### Contaminated site and soil microbial culture generation

A complex microbial community was retrieved from the shallow fraction (0 to 1.2 meter depth) of a long-term CE-contaminated soil, sampled with a pedological needle on the right bank of the river Ploučnice in SAP Mimoň *(*Czech Republic, 50°37′55.353″N, 14°43′16.401″E). The site was contaminated by the leakage of tetrachloroethylene used during improper management of animal waste. Before the preparation of microbial cultures, the contaminated soil was aerated overnight in a fume hood to facilitate the evaporation of highly volatile CEs. At the preliminary stage of the present study, the capacity of the initial soil microbial community to degrade cDCE was investigated. Microbial suspensions were prepared as 10% (w/v) soil dilutions in MSM in order to minimize initial cDCE concentration levels. Each suspension was spiked with ∼0.35 µmol of cDCE, corresponding to a concentration of ~7 µmol∙L^−1^ in the aqueous phase. Depletion of cDCE was regularly evaluated over a time frame of 10 days and only once in two weeks up to a period of three months.

### Cultivation on toluene and phenol

A 10% (w/v) soil suspension was prepared in MSM without replication, incubated for 24 hours and shaken at 130 rpm at 20 °C. This suspension was then used as an inoculum (25% v/v in fresh MSM) for the preparation of microbial cultures which were exposed to 20 µmol∙L^−1^ of toluene or phenol with 0.5 mg∙L^−1^ yeast extract. These cultures were spiked with ∼0.35 µmol of cDCE per vial initially, corresponding to a concentration of ~7 µmol∙L^−1^ in the aqueous phase. The cDCE depletion was monitored over time.

### Cultivation on SPMEs

Microbial cultures were separately established in the same way as cultures exposed to toluene or phenol but were amended with one of the following eight monoaromatic SPMEs as sole carbon sources: salicylic acid, acetophenone, phenethyl alcohol, *p*-hydroxybenzoic acid, *trans*-cinnamic acid, ferulic acid, vanillic acid and caffeic acid. An initial concentration of 50 mg∙L^−1^ of each SPME (equivalent to molar concentration values between 0.3 and 0.4 mM) was supplied to each culture along with a spike of ∼0.25 µmol of cDCE, corresponding to an initial concentration of ~5 µmol∙L^−1^ of cDCE in the aqueous phase. No biological replicates were used in this initial screening experiment. The cultures described above were kept for a period of 33 days and were newly propagated into fresh medium containing SPME and cDCE each time cDCE was completely depleted. Four microbial cultures exhibiting higher cDCE depletion rates over time were selected for further enrichment on SPMEs and cDCE-degradation experiments.

### SPME-based liquid enrichment cultures

Each culture selected was used to inoculate new enrichment cultures in three replicates (25% v/v into fresh MSM), which were propagated every 10 days. A total of seven propagation steps were performed. At the beginning of each culturing step, each vial was provided with 50 mg∙L^−1^ of each SPME and was spiked with ∼0.25 µmol cDCE (corresponding to an initial concentration of ~5 µmol∙L^−1^ in the aqueous phase). SPME and cDCE depletions were monitored over time. Microbial growth was monitored through protein content in microbial cell pellets obtained from 4 mL of culture. The pellets were further resuspended in 0.5 mL of deionized water adjusted with 0.1 M NaOH and boiled for 1 minute. The protein concentration (mg∙L^−1^) was measured according to the Folin-Ciocalteau reagent-based method^66^, the test colorimetric reaction was conducted in the presence of 0.1 M NaOH and quantification was based on bovine serum albumin standard solutions.

### Depletion of cDCE in the selected SPME-enriched cultures

The cDCE transformation yield was calculated as the ratio between total cDCE depleted (µmol) and total SPME consumed (mmol) in each vial during the 10 days of cultivation.

### Analytical methods used to quantify SPMEs and cDCE

The concentration of SPMEs in the MSM was determined by means of High Performance Liquid Chromatography (HPLC) on a HP1100 device (Agilent) equipped with a Luna column (C18-2, 150 × 2 mm, 5 µm, Phenomenex) connected to a diode array detector. Analytical conditions were as follows: a mobile phase flow of 0.4 mL∙min^−1^; isocratic elution; injection volume of 10 µl. The mobile phase was constituted from 0.1% H_3_PO_4_ acidified analytical water and methanol in a 3:1 ratio for the detection of *p*-hydroxybenzoic acid, 1:1 for *trans*-cinnamic acid and 3:2 for acetophenone and phenethyl alcohol. The cDCE was measured by sampling 200 μL of the headspace with a gas-tight syringe (Hamilton) which was then injected into a GC-µECD Agilent 6890 N instrument. The concentration of cDCE (µmol∙L^−1^) in the aqueous phase was deduced according to Henry’s law while assuming equilibrium between the liquid and gas phases. The dimensionless Henry coefficient for cDCE at 20 °C was 0.127, as indicated by U.S. E.P.A. On-line Tools for Site Assessment Calculation (https://www3.epa.gov/ceampubl/learn2model/part-two/onsite/esthenry.html). Standard solutions of cDCE were prepared in MSM with the same liquid-to-headspace gas ratio as experimental vials. The GC instrument was equipped with an electron capture detector and a capillary column (DB-XLB, J&B, 30 m, 0.25 mm, film 0.25 µm, mobile phase: nitrogen). The analytical conditions were as follows: nitrogen flow, 165 ml ∙ min^−1^; speed, 59 cm ∙ s^−1^; injection chamber temperature, 135 °C; column temperature, 135 °C; detector temperature, 250 °C; split ratio, 30:1; time analysis, 2 min. The microbial cultures, which were previously grown to reach microbial concentrations comparable to those obtained in the active cultures but inhibited with 1 g∙L^−1^ sodium azide, were used to evaluate possible losses of cDCE through non-biological processes.

### DNA isolation and 16S rRNA gene sequencing

Total metagenomic DNA was isolated from the triplicate samples of the initial contaminated soil and from the SPME-enriched microbial cultures (three biological replicates per each treatment) obtained at the end of the enrichment procedure. DNA extraction was performed using the FastDNA Spin Kit for Soil (MP Bio, USA) according to the manufacturer’s instructions. Primers 515 forward 5′-GTGYCAGCMGCNGCGG-3′ and 926 reverse 5′-CCGYCAATTYMTTTRAGTTT-3′ (adapted from Walters, *et al*.^67^ with modification of the forward primer) were used to target hypervariable regions V4–V5 of the 16S rRNA genes amplified by PCR in a final volume of 15 µL with: KAPA HiFi HotStart ReadyMix (Kapa Biosystems, Boston, MA, USA) containing 0.02 U/µL of KAPA HiFi HotStart DNA Polymerase, 2.5 mM MgCl_2_ and 0.3 mM of each dNTP; 0.3 µM of each primer (Generi Biotech, Czech Republic); and template DNA (~20 ng). The cycling program was set as follows: 5 min at 95 °C, 20 cycles of 20 s at 98 °C, 15 s at 56 °C, 15 s at 72 °C and a final extension of 5 min at 72 °C. A volume of 0.5 µL of the PCR product was used as the template for another round of PCR, which was performed under the same conditions except that the final reaction volume was 25 µL, with 1 µM of each primer, and the cycle number was decreased to 5-10. The forward and reverse primers used for the second PCR were modified with sequencing adapters and internal barcodes of variable length (5–8 bp) using the TaggiMatrix spreadsheet courtesy of Travis C. Glenn at the University of Georgia (http://www.baddna.org). The resultant PCR products were purified using AMPure XP Beads (Agencourt, Beckman Coulter, USA). Further amplicon-sample library preparation and sequencing analysis on an Illumina MiSeq instrument were performed at the Core Facility for Nucleic Acid Analysis at the University of Alaska Fairbanks.

### Sequencing data analysis

A pipeline for the analysis of amplicon sequences was adapted from Callahan, *et al*.^68^ using DADA2. The slight modifications made to the DADA2 SOP resulted from the analysis of a mock community, which consisted of 12 bacterial strains and was amplified along with the sample sequences. Briefly, octuplicate sequences for each biological replicate (fastaq files) were filtered according to their quality (parameters truncLen = c(260,260), maxN = 0, maxEE = 2, truncQ = 2), while the primer sequences were trimmed off to the final degenerated position. Upon dereplication, a DADA2-based removal of sequencing errors was performed, followed by the merging of the denoised forward and reverse reads and the removal of chimeric sequences. Based on the results of mock community analysis, sequences differing by one base were clustered together, with the most abundant sequence being regarded as correct; octuplicate sequences were merged, while keeping only those sequences that occurred in at least three of the octuplicates. Finally, taxonomy was assigned to the valid sequences using rdp_train_set_14^69^ to create a table of sequence variants.

Further sequence analyses were carried out in R project^70^ using the phyloseq package^71^, with diversity indexes being calculated on the basis of rarefied data (14,000 valid reads); ordination analysis was carried out on CSS-normalized data^72^, while abundance barcharts were constructed from merged biological replicates. The structural diversity of the community was described by the number of sequence variants observed, Simpson’s Index of Diversity (1–*D*), Simpson’s reciprocal index (1/*D*) and the Shannon index^73, 74^. The Bray-Curtis distance-based unconstrained ordination method for NMDS was used to evaluate the ecological distance between the four enriched microbial cultures.

### Sequence deposition

MiSeq reads were deposited in the NCBI Short Read Archive under SRA study number SRP090758.
